# FGL1 regulates acquired resistance to Gefitinib by inhibiting apoptosis in non-small cell lung cancer

**DOI:** 10.1186/s12931-020-01477-y

**Published:** 2020-08-10

**Authors:** Cuilan Sun, Weiwei Gao, Jiatao Liu, Hao Cheng, Jiqing Hao

**Affiliations:** 1 Department of Oncology, The First Affiliated Hospital of Anhui Medical University, Hefei, 230022 Anhui China; 2 Department of Pharmacy, the First Affiliated Hospital of Anhui Medical University, Hefei, China

**Keywords:** Fibrinogen-like-protein 1, Epidermal growth factor receptor, Non-small cell lung cancer, Gefitinib resistance, Apoptosis

## Abstract

**Background:**

This study investigated the role of fibrinogen-like protein 1 (FGL1) in regulating gefitinib resistance of PC9/GR non-small cell lung cancer (NSCLC).

**Methods:**

The effect of different concentrations of gefitinib on cell proliferation were evaluated using the CCK-8 assay. FGL1 expression in the normal human bronchial epithelial cell line Beas-2B, as well as four lung tumor cell lines, H1975, A549, PC9, and PC9/GR, was investigated by using western blotting and qRT-PCR. *FGL1* was knocked down using small interfering RNA to evaluate the effects of FGL1 on PC9 and PC9/GR. The correlation between FGL1 expression and gefitinib resistance was determined in vitro via CCK-8 and colony formation assays, and flow cytometry and in vivo via flow cytometry and immunohistochemistry*.*

**Results:**

FGL1 expression was significantly upregulated in non-small cell lung cancer cells with EGFR mutation and higher in the gefitinib-resistant NSCLC cell line PC9/GR than in the gefitinib-sensitive NSCLC cell line PC9. Further, FGL1 expression in PC9 and PC9/GR cells increased in response to gefitinib treatment in a dose-dependent manner. Knockdown of *FGL1* suppressed cell viability, reduced the gefitinib IC50 value, and enhanced apoptosis in PC9 and PC9/GR cells upon gefitinib treatment. Mouse xenograft experiments showed that *FGL1* knockdown in PC9/GR tumor cells enhanced the inhibitory and apoptosis-inducing actions of gefitinib. The potential mechanism of gefitinib in inducing apoptosis of PC9/GR cells involves inhibition of PARP1 and caspase 3 expression via suppression of FGL1.

**Conclusions:**

FGL1 confers gefitinib resistance in the NSCLC cell line PC9/GR by regulating the PARP1/caspase 3 pathway. Hence, FGL1 is a potential therapeutic target to improve the treatment response of NSCLC patients with acquired resistance to gefitinib.

## Background

Lung cancer is the main cause of cancer-related mortality globally [1]. In China, 7,33,300 lung cancer cases were diagnosed in 2015 [2]. Non-small cell lung cancer (NSCLC) accounts for approximately 85% of lung cancer cases, and approximately 80% of NSCLC patients miss the best opportunity of treatment by the time of diagnosis. With a five-year survival rate of only 15%, prognosis is poor [3]. The frequency of epidermal growth factor receptor (EGFR) gene mutation in non-smoking NSCLC patients is as high as 60% in Asia [4]. Especially abnormal *EGFR* activation can promote the progression of NSCLC [5].

EGF receptor tyrosine kinase inhibitors (EGFR-TKIs) are currently used as the first-line treatment in advanced NSCLC patients harboring *EGFR* mutation [6, 7]. Although these TKIs have good initial efficacy, approximately 65% of EGFR-TKI-sensitive NSCLC patients eventually develop acquired resistance to these drugs after 9–13 months of treatment [8, 9]. The resistance to EGFR-TKI can be primary or acquired. The mechanisms of primary drug resistance include *KRAS* mutation and different *EGFR* mutation sites inducing different levels of sensitivity. The mechanisms of acquired resistance to EGFR-TKIs include secondary mutation of T790M and C797S in EGFR [10] and activation of signaling pathways downstream of EGFR through BRAF fusion and PIK3CA mutation [11], bypass activation, and cell phenotype transformation [12, 13]. Particularly, the activation of downstream and bypass signaling plays an important role in overcoming drug resistance. Further, substantial evidence indicates that numerous cytokines related to cell proliferation play key roles in pathways that promote tumor cell proliferation and suppress their apoptosis [14, 15], thereby significantly affecting patient prognosis. Benefited from the results above, some corresponding inhibitors like MEK inhibitors (trimetazidine) [16, 17], MET-TKIs (tepotinib and cabozantinib) [18, 19], PI3K inhibitor [20], and STAT3 and Src inhibitors [21, 22] have been developed widely applied in clinical and showing good clinical effects. Some newly discovered cytokines, including YES (pp62c-yes) [23], YES/YES-associated protein 1 [24], and NF-1 [25], can increase the sensitivity of NSCLC cells to EGFR-TKIs by activating the AKT or MAPK pathway, showing great research benefits. However, in 20–30% of cases of acquired resistance, the mechanism underlying resistance development remains unclear [26, 27]. Thus, numerous studies have focused on the underlying mechanism of acquired resistance to EGFR-TKIs in NSCLCs. It is well known that one of the important mechanisms of gefitinib resistance in NSCLCs is the activation of downstream or bypass pathways of cell growth and proliferation through certain unknown and key cytokines.

Fibrinogen-like protein 1 (FGL1), a member of the fibrinogen family, is a specific hepatocyte mitogen [28, 29]. FGL1 regulates proliferation factor expression, promotes liver regeneration, and repairs liver damage [30–32]. Recently, FGL1 overexpression has been reported in many solid tumors, especially in NSCLC, and was associated with shorter 5-year overall survival [7]. Studies have shown that bone marrow stromal cells (BMSCs) overexpress FGL1 to repair acute liver injury by regulating p-STAT/STAT3 [33], and overexpression of FGL-1 was associated with epithelial intermediate transformation and angiogenesis of *LKB1*-mutant lung adenocarcinoma cells [34]. FGL1 has also been reported to regulate mitochondrial activity and oxidative phosphorylation, which are related to cell growth and proliferation. This may be mediated by EGFR activation via direct phosphorylation of EGFR or through non-receptor tyrosine kinase SRC, which activates the ERK/p-ERK pathway to promote cell proliferation [22]. Importantly, FGL1 expression not only affects the regeneration of hepatocytes, but may also regulate the growth and proliferation of tumor cells due to its role in cell proliferation pathways. However, the possible role of FGL1 in regulating NSCLC cell proliferation and acquired resistance to gefitinib has not been reported to date.

In the present study, we used the NSCLC cell line PC9 and the gefitinib-resistant PC9 cell line PC9/GR to investigate the role of FGL1 in acquired resistance to gefitinib in NSCLC. Our results show that FGL1 is a potential target for overcoming EGFR-TKI resistance in NSCLC patients.

## Methods

### Cells and culture conditions

The NSCLC cell line PC9 and the gefitinib-resistant PC9 cell line PC9/GR were purchased from the cell bank of the Chinese Academy of Sciences (Shanghai, China). BEAS-2B, A549, and H1975 cells, originally purchased from the cell bank of the Chinese Academy of Sciences, were provided by the Department of Immunology, Anhui Medical University. BEAS-2B, A549, and PC9 cells were cultured in high-glucose DMEM medium (SH30022.01B; HyClone, Beijing, China) supplemented with 10% fetal bovine serum (11011–8611; Sijiqing Biotechnology, Hangzhou, China) and 1% penicillin-streptomycin (3810-74-0; Sigma, USA) at 37 °C in the presence of 5% CO_2_. Gefitinib (MB1112; Meilune, Dalian, China) was added to the culture medium at a concentration of 0.1 μmol/L to sustain the drug resistance phenotype of PC9/GR cells. H1975 cells were cultured in RPMI 1640 medium (SH30809.01B; HyClone, USA).

### Small interfering (si)RNA transfection

*FGL1* expression was knocked down using siRNAs designed at GenePharma (Shanghai, China). The target sequences were as follows: FGL1-siRNA1, GGAGGAGGAUGGACUGUAATT; FGL1-siRNA2, GCCGUUAUGCACAAUAUAATT; FGL1-siRNA3, GCAAACCUGAAUGGUGUAUTT. Blank siRNA was used as a control (NC-siRNA). Cells were seeded in 6-well plates (1.0 × 10^5^ cells/ml) and cultured for 24 h. When the cells reached 40–60% confluence, they were transfected with the siRNAs in accordance with the instructions of the Lipofectamine™ 2000 kit (11668–027; Invitrogen, USA). Non-treated PC9/GR cells were included as a control group. Then, the cells were treated with gefitinib (gefitinib and gefitinib+FGL1-siRNA groups). After 48 h of transfection, total RNA was extracted using TRIzol reagent (R4801–01; Magen, Beijing, China). *FGL1* knockdown was verified by RT-qPCR and western blotting. FGL11-siRNA2 and FGL1-siRNA3 produced the most stable interference effects in tests conducted at Shanghai Jikai Company and were selected for use in experiments.

### qRT-PCR

Total RNA was isolated from PC9/GR tumors collected from mice (details on the mice used and ethical clearance of the study are given in a section below) and NSCLC cells using TRIzol reagent and reverse-transcribed into cDNA using the PrimeScript™ One Step RT-PCR kit (RR036A; Takara, Japan). PCRs were run using TB Green™ Premix Ex Taq™ II (RR820A, Takara) on a LightCycler96 PCR (Roche, USA). *GAPDH* was used as internal control to normalize relative gene expression by the 2^–∆∆^ CT method.

### Cell viability assay

Stably transfected PC9 or PC9/GR cells were seeded into 96-well plates at 2.5 × 10^4^ cells/well and cultured for 24 h. Then, the cells were cultured in the presence of different concentrations of gefitinib (0, 0.6125, 1.25, 2.5, 5, 10, 20, and 40 μmol/L) for 48 h. CCK-8 reagent (BB-4202-500 T; BestBio, Nanjing, China) was added (10 μl/well) and the plate was incubated for another 2 h. The absorbance (A) at 450 nm was measured using a microplate reader (Stat Fax-4200, USA), and the cell survival rate was calculated by using the following formula: cell survival rate (%) = [(administration group A – negative control group A)/(non-administration group A – Negative control group A)] × 100%. The half-maximal inhibitory concentration (IC50) was calculated based on the relative survival curve using GraphPad Prism v. 7.0 (GraphPad Software, CA, USA).

### Apoptosis detection by flow cytometry

Stably transfected PC9 cells and PC9/GR cells were seeded in 6-well plates at 3 × 10^5^ cells/well and cultured in the presence of different concentration of gefitinib (0.2 or 8 μmol/L) for 48 h. Purified removed tumor cells were adjusted to 1 × 10^6^/L. The apoptotic rates of NSCLC cells and tumor tissues were evaluated by flow cytometry (FACScan, BD Bioscience) using an Annexin-V-FITC/PI apoptosis kit (ads5001; Absin, Shanghai, China).

### Colony formation assay

Transfected PC9 and PC9/GR cells were seeded in 6-well plates at 1 × 10^3^ cells/well and cultured in the presence of different concentrations of gefitinib (0.2 or 5 μmol/L). The medium was replaced every 3 days for 2 weeks. Then, the cells were fixed with 4% paraformaldehyde (BL539A; Biosharp, Shanghai, China) and stained with 0.1% crystal violet (46364-250MG; Sigma-Aldrich). Colonies containing more than 50 cells were counted randomly under a light microscope (DM3000; Leica, Germany).

### Western blot analysis

Stably transfected PC9 cells and PC9/GR cells were seeded in 6-well plates at 3 × 10^5^ cells/well and cultured for 48 h. The cells were lysed in RIPA buffer containing 1% PMSF. A BCA protein kit (PC0020; Beijing Solabo) was used to determine the protein concentration. The protein extracts were separated by 10% SDS-PAGE, transferred onto PVDF membranes (ISEQ00010; Millipore, USA) and then probed with specific antibodies against FGL1 (ab197357; Abcam, USA), EGFR (26,462,646; Cell Signaling Technology, USA), p-EGFR Y1173 (ab5644; Abcam), p-EGFR Y1068 (ab5644; Abcam), PARP1 (ab4830; Abcam), caspase 3 (ab13847; Abcam), and β-actin (60008–1-Ig; Proteintech, Wuhan, China). After incubation with HRP-coupled secondary antibody, the protein bands were detected in an ECL Advance Detection System (Amersham Biosciences, USA) using a SuperSignal West Femto Tril Kit (34,094; Thermo USA). The gray-scale value of all bands was analyzed using the ImageJ software.

### Lentivirus infection

Lentivirus harboring FGL1-siRNA2 was generated by GeneChem Co., Ltd. (Shanghai, China). Briefly, PC9/GR cells (1 × 10^5^) were seeded into a 6-well plate. When they reached 20% confluence, they were transfected with lentivirus carrying the siRNA and empty control lentiviral vector at MOI value of 20 (1 × 10^7^ virus particles). The lowest lethal concentration of puromycin in PC9/GR cells in the control group was screened by adding puromycin at 0.25, 0.5, 1, or 2 μg/mL. After 48 h of puromycin treatment, the lowest drug concentration causing death of the control cells was 1 μg/mL. Total RNA was extracted from the cells using TRIzol reagent, and effective knockdown was verified by RT-qPCR and western blotting. FGL1-siRNA2, which produced the best interference effect, was used for animal experiments.

### Nude mouse xenograft model

Female BALB/c nude mice (4–5 weeks of age and weighing 16–20 g) were purchased from the experimental animal center of Chinese Academy of Sciences (Shanghai, China) and were acclimatized for 1 week. The mice were maintained in a specific pathogen-free environment and were given free access to standard chow and water. PC9/GR cells (1 × 10^7^) stably transfected with FGL1-siRNA or NC-siRNA were subcutaneously inoculated into the right flanks of the mice (*n* = 6 mice in each group) to establish a lung adenocarcinoma model to investigate the effect of FGL1 on cell proliferation in vivo. When the average tumor volume reached 50 mm^3^, half of the mice in each group were administered gefitinib (30 mg/kg), while the other half was administered the same volume of PBS by oral gavage every day. Every 3 days, the mice were weighed and tumor sizes were assessed with a digital caliper. Tumor volume was calculated according to the formula: V = (a × b^2^)/2, where a and b are the maximal and minimal diameters in millimeters, respectively. Twenty-one days later, the mice were killed and tumors were weighed immediately. All animal experiments were performed with the approval of the Research Ethic Committee and conducted according to the institutional guidelines of the Animal Care and Use Committee at the First Affiliated Hospital of Anhui University.

### Immunohistochemistry

The resected tumor tissues were soaked in formalin and dehydrated, paraffin-embedded, and sectioned at 4 μm thickness. The sections were deparaffinized, hydrated, and microwaved for antigen removal. H_2_O_2_ (3%) was used to eliminate endogenous peroxidase activity. After incubation in 5% bovine serum albumin (A1128; Gentihold, Beijing, China) for 20 min to block non-specific binding, the sections were incubated with primary antibody (diluted at 1:200) at 4 °C overnight followed by a biotinylated secondary antibody (diluted at 1:500) at 37 °C for 60 min. Then, the sections were stained with diaminobenzidine and counterstained with hematoxylin. Finally, all tissue sections were incubated in alcohol and xylene. The sections were observed under an inverted fluorescence microscope (Olympus, Japan) and photographs were acquired in five random fields (magnification, 400×) of each sample. Stained cells were counted, and the positive stain rate was analyzed using ImageJ.

### Statistical analysis

Data were analyzed using SPSS 22.0 statistical software. All experiments were repeated at least thrice. The experimental data are expressed as the mean ± standard deviation (SD). Students t-test ‘was used to compare the means of two groups of independent samples, and results with *P* < 0.05 were considered statistically significant.

## Results

### Effects of gefitinib on the proliferation of A549, H1975, PC9, and PC9/GR cells

**Fig. 1:**
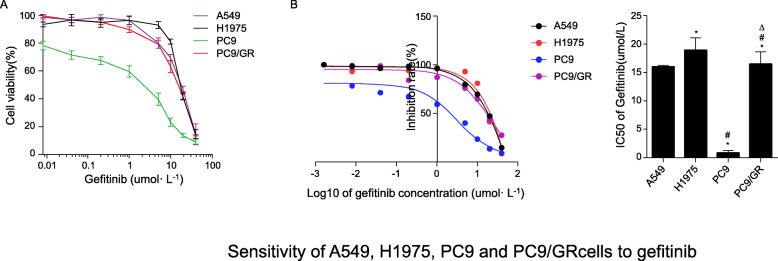
Sensitivity of A549, H1975, PC9, and PC9/GR cells to gefitinib (x ± s, *n* = 5). **a** Inhibition of cell proliferation of A549, H1975, PC9, and PC9/GR cells by gefitinib as measured by CCK-8 assays. **b** IC50 values of gefitinib in A549, H1975, PC9, and PC9/GR cells, calculated using GraphPad. Students t-test‘was used to compare the means of IC50 values of A549, H1975, PC9, and PC9/GR cells by gefitinib. *P < 0.05 compared with A549; #*P* < 0.05 compared with H1975; △*P* < 0.05 compared with PC9

To probe the sensitivity of different NSCLC cell lines to gefitinib, four NSCLC cell lines having a different EGFR status, including A549 (wild-type EGFR), H1975 (L858R and T790M mutation in EGFR exon 21-, PC9 (EGFR exon 19 deletion), and PC9/GR (gefitinib acquired resistant PC9 cells), were exposed to various concentrations of gefitinib (0, 0.625, 1.25, 2.5, 5, 10, 20 and 40 μmol/L) for 48 h. As shown in Fig. 1a, CCK-8 analysis disclosed that cell ability to PC9 cells significantlyl reduced in a concentration-dependent manner upon gefitinib treatment, whereas it had a minimal effect on cell viability at concentrations lower than 1.0 μmol/L. Moreover, cell viability was higher in the other gefitinib-resistant NSCLC cell lines (A549, H1975 and PC9/GR) than in PC9 cells. The IC50 values of the above-mentioned four NSCLC cells for gefitinib were calculated and shown in Fig. 1b and c, were 18.90 μmol/L, 16.40 μmol/L, 1.794 μmol/L and 15.99 μmol/L for A549, H1975, PC9, and PC9/GR, respectively (*P* < 0.05). Take together, these data indicated that the four NSCLC cell lines have different sensitivity to gefitinib, with PC9 cells being the most sensitive.

### Upregulation of FGL1 correlates with gefitinib resistance

**Fig. 2:**
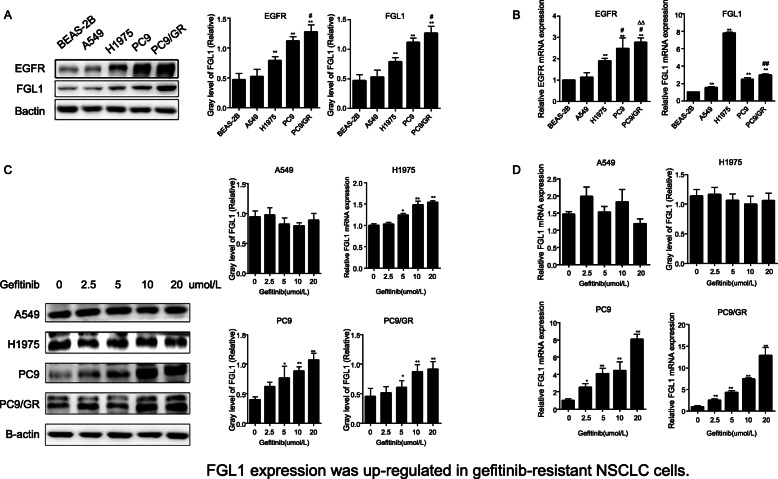
FGL1 expression is upregulated in gefitinib-resistant NSCLC cells (x ± s, n = 5). **a** Protein levels of EGFR and FGL1 in BEAS-2B, A549, H1975, PC9, and PC9/GR cells as measured by western blotting. **b** mRNA expression of *EGFR* and *FGL1* in BEAS-2B, A549, H1975, PC9, and PC9/GR cells as measured by RT-qPCR. **c** Western blot analysis of FGL1 in A549, H1975, PC9, and PC9/GR cells treated with gefitinib. **d** mRNA level of *FGL1* in A549, H1975, PC9, and PC9/GR cells treated with gefitinib as detected by RT-qPCR. The means of protein and mRNA levels of EGFR and FGL1 in BEAS-2B, A549, H1975, PC9, and PC9/GR cells was compared by Students t-test‘. *P < 0.05 compared with A549; #*P* < 0.05 compared with H 1975; △*P* < 0.05 compared with PC9

To explore the role of FGL1 in NSCLC cell resistance to gefitinib, we first measured FGL1 expression levels in normal bronchial epithelial cells BEAS-2B, and the four human NSCLC cell lines, A549, H1975, PC9, and PC9/GR, by western blot analysis and RT-qPCR. As shown in Fig. 2a, FGL1 expression was significantly higher in tumor cells (except A549 cells) than in normal BEAS-2B cells (*P* < 0.01), and among the four tumor cell lines, PC9 and PC9/GR cells exhibited higher FGL1 protein levels than A549 and H1975 cells (*P* < 0.05). The RT-qPCR results were consistent with the western blot data. These results suggested that FGL1 expression may relate to the mutation status of EGFR and contribute to gefitinib acquired resistance in NSCLC cells. To investigate whether FGL1 plays a role in the acquired resistance of NSCLC cells to EGFR-TKIs, we treated A549, H1975, PC9, and PC9/GR cells with gefitinib at various concentrations (0, 0.625 1.25, 2.5, 5, 10, 20, and 40 μmol/L). The results showed that gefitinib significantly increased FGL1 expression in PC9 and PC9/GR cells at both the mRNA and protein levels, in a concentration-dependent manner (*P* < 0.05) (Fig. 2b), whereas it had a minimal effect on FGL1 expression in A549 and H1975 cells (only mRNA expression increased in a concentration-dependent manner in H1975; *P* < 0.05). Together, these results indicated that the upregulation of FGL1 may be correlated with gefitinib acquired resistance in NSCLC cells with EGFR mutation.

### Knockdown of *FGL1* expression overcomes acquired resistance to gefitinib in PC9/GR cells

**Fig. 3:**
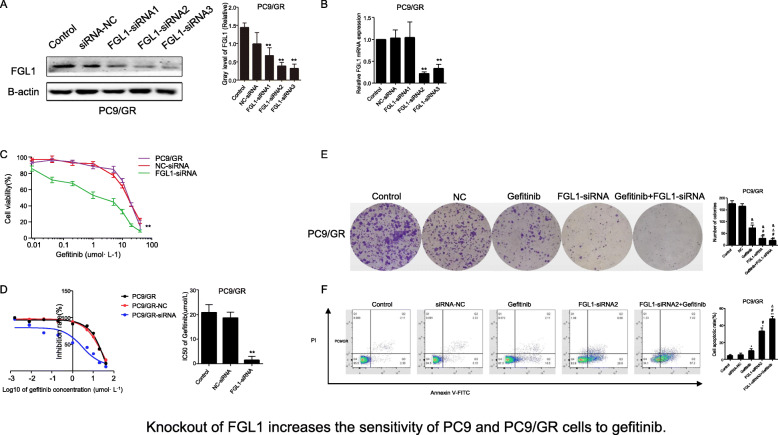
Knockdown of *FGL1* increases the sensitivity of PC9/GR cells to gefitinib in vitro (x ± s, *n* = 3). **a** Relative expression of FGL1 protein in PC9/GR cells after FGL1 interference as measured by western blotting. **b** Relative expression of *FGL1* mRNA in PC9/GR cells after FGL1 knockdown as measured by RT-qPCR. **c** Proliferation of PC9/GR cells inhibited by gefitinib after *FGL1* knockdown as measured by CCK-8. **d** Comparison of IC50 values of gefitinib in PC9/GR cells after *FGL1* knockdown. **e** Colony-forming potential was assessed in PC9/GR cells. **f** Apoptotic rate of PC9/GR cells as evaluated by flow cytometry. Control: blank control group; NC-siRNA: mice transfected with NC-siRNA; Gefitinib: mice treated with gefitinib alone; FGL1-siRNA: mice treated with FGL1-siRNA-transfected cells; gefitinib+FGL1-siRNA: mice transfected with FGL1-siRNA and treated with gefitinib; *P < 0.05 compared with control group; **P < 0.01 compared with control group; #*P* < 0.05 compared with gefitinib group; (*P* < 0.05 compared with gefitinib group; △*P* < 0.01 compared with FGL1-siRNA group; &*P* < 0.01 compared with PC9 cells

To investigate the effects of FGL1 on acquired resistance to gefitinib in NSCLC cells, we knocked down *FGL1* in PC9/GR using siRNA. After siRNA transfection for 48 h, FGL1 expression was strongly reduced at both the mRNA and protein levels (Fig. 3a, *P* < 0.01). Next, we treated the FGL1-knockdown PC9/GR cells with gefitinib at different concentrations (0, 0.625, 1.25, 2.5, 5, 10, 20, and 40 μmol/L) for 48 h. CCK-8 assays showed that downregulation of FGL1 significantly reduced cell viability and lowered the IC50 values (1.445 ± 0.617 μmol/L vs. 18.716 ± 2.167 μmol/L vs. 20.865 ± 3.164 μmol/L) in PC9/GR cells in response to gefitinib treatment as compared to non-transfected PC9/GR cells (Fig. 3b and c, *P* < 0.01). Flow cytometry (Fig. 3d) and colony-formation assays (Fig. 3e) revealed that *FGL1* knockdown enhanced apoptosis and suppressed the colony number of PC9/GR cells, and even led to a considerable increase in gefitinib-induced apoptosis and a substantial decrease in colony number in PC9/GR cells treated with gefitinib (*P* < 0.05). These results suggested that knockdown of FGL1 increased the acquired resistance to gefitinib in PC9/GR cells, and overexpression of FGL1 contributed, at least in part, to apoptosis induced by gefitinib in PC9/GR cells in vitro*.*

### Knockdown of FGL1 enhances the antitumor effects of gefitinib in vivo

**Fig. 4:**
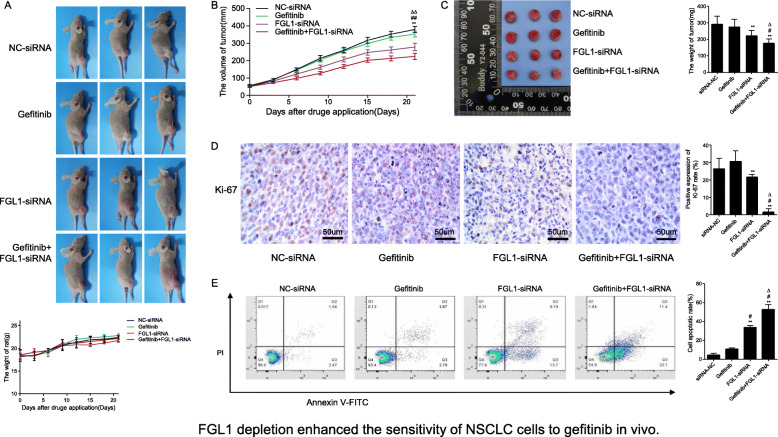
*FGL1* depletion enhances the sensitivity of NSCLC cells to gefitinib in vivo (x ± s, *n* = 3). **a** Mice weight was monitored every three days in the different treatment groups. **b** Tumor size was monitored every three days in the different treatment groups. **c** Tumor weight was monitored in the different treatment groups. **d** Protein expression of Ki-67 in tumor tissues of mice of the different treatment groups as measured by immunohistochemistry. **e** Apoptosis in tumor tissues in each group as analyzed by flow cytometry. Control: blank control group; NC-siRNA: mice transfected with NC-siRNA; Gefitinib: mice treated with gefitinib alone; FGL1-siRNA: mice treated with FGL1-siRNA-transfected cells; gefitinib+FGL1-siRNA: mice transfected with FGL1-siRNA cells and treated with gefitinib. *P < 0.05 compared with control group; **P < 0.01 compared with control group; #*P* < 0.05 compared with gefitinib group; △*P* < 0.01 compared with FGL1-siRNA group

To investigate the effects of FGL1 on the sensitivity of PC9/GR cells to gefitinib in vivo, a xenograft mouse model was established by subcutaneously injecting PC9/GR cells that had been transfected with lentivirus harboring NC-siRNA or FGL1-siRNA into BALB/c nude mice. When the average tumor volume reached 50 mm^3^, gefitinib was given daily at 30 mg/kg by oral gavage, after which tumor volume and body weight were monitored every other day. As shown in Fig. 4a, there was no significant difference in body weight between the groups (*P* > 0.05). However, tumor volumes (Fig. 4b, *P* < 0.05) and tumor weights (Fig. 4c, *P* < 0.05) were significantly lower in FGL1-siRNA-treated mice and in mice treated with FGL1-siRNA and gefitinib than in mice treated with NC-siRNA or gefitinib alone. Immunohistochemistry of the tumor tissues revealed that knockdown of *FGL1* alone or co-culture with gefitinib significantly decreased the Ki-67 levels (Fig. 4d, *P* < 0.05). Flow-cytometric analysis of cells isolated from freshly isolated tumor tissues revealed that both *FGL1* knockdown alone and *FGL1* knockdown plus with gefitinib significantly increased the apoptotic rate (Fig. 4e, *P* < 0.05). Collectively, these results suggested that FGL1 knockdown suppresses tumor growth and significantly enhances the antitumor effect of gefitinib in vivo.

### Knockdown of FGL1 overcomes acquired resistant to gefitinib via activating apoptotic pathway

**Fig. 5:**
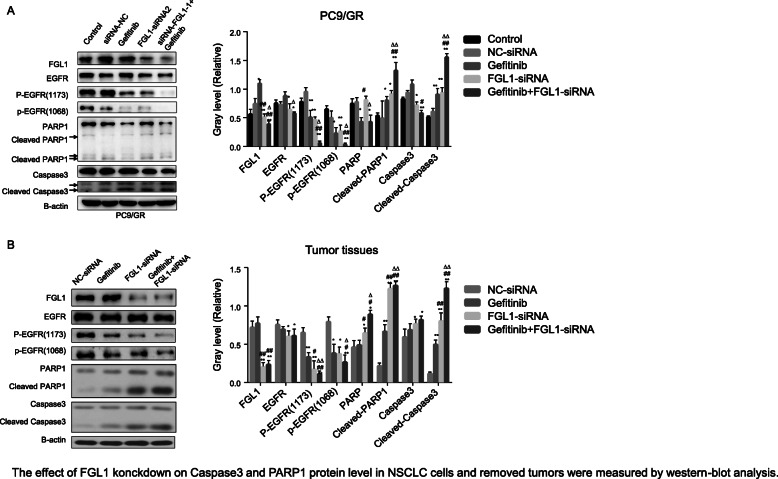
Effect of *FGL1* knockdown on caspase 3 and PARP1 protein level in PC9/GR cells and removed tumors measured by western blot analysis (x ± s, n = 3). **a** Western blot analysis was applied to determine the effect of FGL1 on EGFR, p-EGFR, caspase 3, and PARP1 protein in NSCLC acquired-resistant cell line PC9/GR. **b** Western blot analysis was applied to determine the effect of FGL1 on EGFR, p-EGFR, caspase 3 and PARP1 protein expression in tumor tissues. Control: blank control group; NC-siRNA: mice transfected with NC-siRNA; Gefitinib: mice treated with gefitinib alone; FGL1-siRNA: mice treated with FGL1-siRNA-transfected cells; gefitinib+FGL1-siRNA: mice transfected with FGL1-siRNA cells and treated with gefitinib. *P < 0.05 compared with control group; **P < 0.01 compared with control group; #*P* < 0.05 compared with gefitinib group; △*P* < 0.01 compared with FGL1-siRNA group; &*P* < 0.01 compared PC9 cells

To clarify the potential mechanisms by which FGL1 mediates gefitinib acquired resistance, we measured the expression of proteins related to apoptosis and proliferation in tumor cells by western blot analysis. PARP1 is a major member of the PARP family, and it plays an important role in DNA repair and cell death, proliferation, and differentiation as a sensor and signal transducer. Caspase 3 is the most important terminal shear enzyme involved in the process of cell apoptosis; tt recognizes the DEVD motif in the nuclear localization signal of its substrate PARP1 and disrupts the activity of the enzyme. EGFR is activated through phosphorylation. Therefore, we detected p-EGFR(1173) and p-EGFR(1068). As shown in Fig. 5a, knockdown of *FGL1* expression in PC9/GR cells significantly decreased the protein levels of EGFR, p-EGFR(1173), p-EGFR(1068), whereas it decreased the levels of PARP1 and caspase 3, along with increased expression of cleaved PARP1 and cleaved caspase 3 (all *P* < 0.05). We detected the above-mentioned apoptosis-related proteins in tumor tissues freshly collected from mice inoculated with FGL1-siRNA-transfected PC9/GR cells and treated or not with gefitinib. As expected, gefitinib alone inhibited the expression of EGFR, p-EGFR (1173), and p-EGFR (1068), but did not affect the levels of PARP1 and caspase 3, whereas knockdown of FGL1 plus gefitinib treatment not only significantly decreased the protein levels of p-EGFR (Y1173 and Y1068), but also increased cleaved PARP1 and cleaved caspase 3 levels by cleaving PARP1 and caspase 3 (Fig. 5b, all *P* < 0.05). In summary, FGL1 depletion promotes the sensitivity of PC9/GR cells to gefitinib in vitro or in vivo, partly via activation of the PARP1/caspase 3 pathway.

### Inhibition of FGL1 enhances the antitumor effects of gefitinib via inducing apoptosis in PC9 cells

**Fig. 6:**
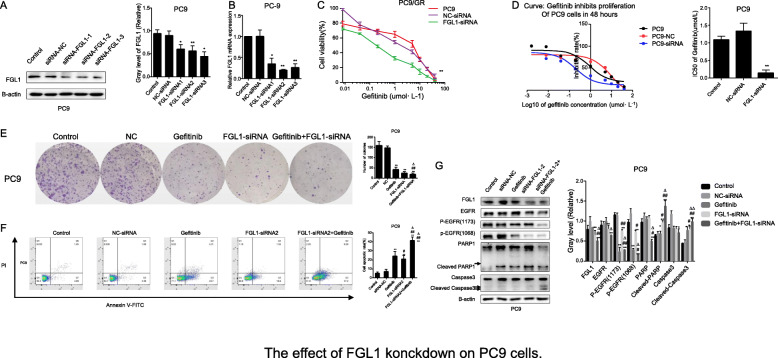
Effect of *FGL1* knockdown on PC9 cells. **a** Relative expression of FGL1 protein in PC9 cells after FGL1 knockdown as measured by western blotting. **b** Relative expression of *FGL1* mRNA in PC9 cells after *FGL1* knockdown as measured by RT-qPCR (x ± s, n = 3). **c** Proliferation of PC9 cells inhibited by gefitinib after *FGL1* knockdown as measured by CCK-8 assy. **d** Comparison of IC50 values of gefitinib in PC9 cells before and after FGL1 interference. **e** Colony-forming potential assessed in PC9 cells and PC9 cells. **f** Apoptotic rate of PC9 cells as evaluated by flow cytometry. **g** Western blot analysis of the effect of FGL1 on EGFR, p-EGFR, caspase 3 and PARP1 protein in the sensitive NSCLC cell line PC9. Control: blank control group; NC-siRNA: mice transfected with NC-siRNA; Gefitinib: mice treated with gefitinib alone; FGL1-siRNA: mice treated with FGL1-siRNA-transfected cells; gefitinib+FGL1-siRNA: mice transfected with FGL1-siRNA cells and treated with gefitinib. *P < 0.05 compared with control group; **P < 0.01 compared with control group; #*P* < 0.05 compared with gefitinib group; △*P* < 0.01 compared with FGL1-siRNA group; &*P* < 0.01 compared PC9 cells

To investigate whether FGL1 has the same apoptosis-promoting effect in gefitinib-sensitive NSCLC cells, we also knocked down FGL1 expression in PC9 cells using siRNA. As shown in Fig. 6a and b, *FGL1* expression was obviously reduced in PC9 cells transfected with siRNA (*P* < 0.05). We found that FGL1 knockdown not only increased the sensitivity to gefitinib of PC9 cells, but also affected their survival in vitro. *FGL1* knockdown significantly reduced cell proliferation (Fig. 6c),IC50 values for gefitinib and colony formation (Fig. 6e) and substantially increased the apoptotic rate (Fig. 6f) (all *P* < 0.05). Furthermore, FGL1 knockdown of significantly suppressed cell proliferation and lowered the IC50 value (0.143 ± 0.085 μmol/L vs. 1.092 ± 0.106 μmol/L) in PC9 cells exposed to gefitinib (Fig. 6c, *P* < 0.05). These data suggested that FGL1 depletion also promotes apoptosis and increases gefitinib sensitivity in PC9 cells. As for the mechanism, as shown in Fig. 6g, proteins related to apoptosis were expressed at the levels observed in PC9/GR cells after *FGL1* knockdown in PC9 cells. Further, *FGL1* knockdown inhibited EGFR and EGFR phosphorylation (p-EGFR1173 and p-EGFR1086), and decreased PARP1 and caspase 3 levels, regardless of the presence of gefitinib (*P* < 0.05), which implied that *FGL1* promotes apoptosis of NSCLC cells PC9 through affecting the expression of PARP1/caspase 3 via lowering the expression and phosphorylation of EGFR.

## Discussion

The development of resistance to targeted therapy with EGFR-TKIs remains a major clinical challenge. In the present study, we found that FGL1 expression is significantly increased in gefitinib-resistant PC9/GR cells, and knockdown of FGL1 enhanced gefitinib-induced apoptosis and inhibited cellular proliferation in PC9/GR cells. Moreover, we found that FGL1 can regulate the phosphorylation level of EGFR and the expression levels of apoptosis-related proteins, such as cleaved caspase 3 and cleaved PARP1. To the best of our knowledge, this is the first study to report the regulatory role of FGL1 on NSCLC growth and acquired resistance to gefitinib, suggesting that FGL1 may be a potential target for NSCLC therapy.

EGFR-TKIs, such as gefitinib and erlotinib, have been widely used in the clinical treatment of NSCLC. However, patients eventually develop resistance due to various mechanisms, such as the occurrence of secondary EGFR mutations (T790M), activation of alternative pathways (c-Met, HGF, AXL), downstream pathway abnormalities (K-ras mutations, PTEN loss), or EGFR-TKI-mediated apoptosis pathway damage. Moreover, the clinical benefits of these TKIs are still limited. Therefore, the precise mechanism of EGFR-TKI resistance should be elucidated.

Numerous studies have revealed that FGL1 is significantly upregulated after liver injury and acts as a factor regulating proliferation, promoting liver regeneration and repairing liver injury by inducing ERK1/2 or STAT3 phosphorylation [31, 32, 35]. Several recent studies have shown that FGL1 is significantly upregulated in NSCLC patients and is closely correlated to the poor prognosis of these patients [7, 31, 34, 36]. Further, FGL1 may be an important factor involved in epithelial-mesenchymal transition by influencing cell-cell adhesion and information transmission [37, 38]. Our previous study also revealed that loss of FGL1 did not induce but inhibited epithelial-mesenchymal transition in PC9/GR cells [39]. More interestingly, Bie and colleagues found that FGL1 affected the proliferation of lung adenocarcinoma cells by regulating the expression of vascular endothelial growth factor, hypoxia-inducible factor, insulin-like growth factor, and EGFR through functional experiments and RNA sequencing [34]. However, no study focused on whether FGL1 is involved in EGFR-TKI in acquired-resistance in NSCLC.

In the present study, we found that gefitinib increased FGL1 expression in gefitinib-sensitive NSCLC cells in a concentration-dependent manner, and gefitinib acquired-resistant PC9/GR demonstrated higher levels of FGL1 than their parental cells. Moreover, loss-of-function experiments revealed that knockdown of FGL1 reduced cell viability and increased apoptosis in PC9/GR cells upon gefitinib treatment both in vitro and in vivo. In line with our findings, Wang and colleagues reported that FGL1 expression was upregulated in NSCLC tissues [33], and FGL1 expression was closely related to the apoptosis of hepatocytes [34]. Taken together, our results suggest that overexpression of FGL1 may contribute to NSCLC cell proliferation and apoptotic resistance, thus leading to EGFR-TKI acquired resistance; however, the exact mechanism remains unclear.

Recent evidence suggests that elevated expression of FGL1 can activate the p-STAT/STAT3 pathway to repair injured hepatocytes through inhibiting apoptosis and promoting proliferation [40], and FGL1 exert an antiapoptotic effect on hepatocytes by inhibiting the upregulation of the apoptotic factors of Bax and caspase-9 and enhancing the expression of the antiapoptotic factors Bcl-2 and Bcl-xl [30, 35]. It has also been shown that STAT3 signaling is involved in the regeneration and apoptosis of liver injury [33, 41, 42], and phosphorylated STAT3 regulated the expression of Bax, Bcl-2, and cell cycle-regulatory genes (including c-fos, c-myc, and cyclin), indicating its role in cell proliferation and apoptosis [43]. Although overexpression of FGL1 has been confirmed in several tumors and contributes to poor prognosis [31, 36–38, 40], FGL1 expression is downregulated in HCC, and loss of FGL1 may lead to the low differentiation of HCC cells [31, 36, 40]. This difference may be closely related to the differentiation of tumor cells [40] and their surrounding microenvironment [28, 38]. In the current study, we found that knockdown of FGL1 led to clearly enhanced the expression of cleaved caspase 3 and cleaved PARP1, and overcame acquired resistance to gefitinib in NSCLC cells both in vitro and in vivo. Similarly, some studies have shown that activation of STAT3/Bcl-2/caspase 3 signaling can promote apoptosis of NSCLC cells [17, 43], and acquired resistance of EGFR-mutated lung cancer to TKI treatment is related to the anti-apoptosis effect of the PARP pathway [44]. Our study unraveled that FGL1 may be involved in the regulation of proliferation and apoptosis in NSCLC cells via modulating the PARP1/caspase 3 pathway.

In addition, high FGL1 expression may be related to the expression level of EGFR, which has been reported in L02 cells and several studies on liver injury [19, 30, 45, 46]. FGL1 can inhibit L02 cell proliferation induced by activating the non-receptor tyrosine kinase SRC to induce EGFR phosphorylation [21]. It has also been observed that apoptosis of NSCLC cells is induced by inhibition of EGFR/STAT3 activation and promotion of PARP1 cleavage, regardless of the mutation status of EGFR [47]. Consistently, we also found that administration of gefitinib and loss of FGL1 suppressed phosphorylation of EGFR (p-EGFR1173 and p-EGFR1086) and promoted apoptosis by reducing the levels of caspase 3 and PARP1 in vitro. However, we used only one lung adenocarcinoma cell line (PC9/GR) for in vitro and in vivo experiments and mouse experiments were not conducted. Therefore, the precise functions of FGL1 in acquired resistance to EGFR-TKIs requires further study. In summary, our results suggest that FGL1 may be an important regulator of EGFR-TKI resistance in NSCLC and targeting FGL1 may be a promising approach to solving the problem of EGFR-TKI acquired resistance.

## Data Availability

All data generated or analyzed during this study are included in this published article [and its supplementary information files].

## Abbreviations

EGFR: Epidermal growth factor receptor
EGFR-TKI: Epidermal growth factor receptor-tyrosine kinase inhibitor
FGL1: Fibrinogen-like protein 1
GAPDH: Glyceraldehyde-3-phosphatedehydrogenase
IC50: Half maximal inhibitory concentration
NC: Negative control
NSCLC: Non-small cell lung cancer
PARP1: PolyADP-ribose polymerase 1
TKI: Tyrosine kinase inhibitor
